# Mitochondrial Mechanisms of Neuroglobin's Neuroprotection

**DOI:** 10.1155/2013/756989

**Published:** 2013-03-24

**Authors:** Zhanyang Yu, Jessica L. Poppe, Xiaoying Wang

**Affiliations:** Neuroprotection Research Laboratory, Departments of Neurology and Radiology, Massachusetts General Hospital, Charlestown, MA 02129, USA

## Abstract

Neuroglobin (Ngb) is an oxygen-binding globin protein that has been demonstrated to be neuroprotective against stroke and related neurological disorders. However, the underlying mechanisms of Ngb's neuroprotection remain largely undefined. Mitochondria play critical roles in multiple physiological pathways including cell respiration, energy production, free radical generation, and cellular homeostasis and apoptosis. Mitochondrial dysfunction is widely involved in the pathogenesis of stroke and neurodegenerative diseases including Alzheimer's, Parkinson's, and Huntington's diseases. Accumulating evidence showed that elevated Ngb level is associated with preserved mitochondrial function, suggesting that Ngb may play neuroprotective roles through mitochondria-mediated pathways. In this paper we briefly discuss the mitochondria-related mechanisms in Ngb's neuroprotection, especially those involved in ATP production, ROS generation and scavenging, and mitochondria-mediated cell death signaling pathways.

## 1. Introduction

Neurological disorders including stroke, brain trauma, and age-related neurodegeneration are leading causes of death and severe long-term disability among adults in the USA and many other countries. The impact of these diseases is devastating in terms of loss of life, decreased life quality for survivors and their families. However, effective therapies are still lacking. To date the only approved therapy against stroke is the thrombolytic therapy using tissue plasminogen activator (tPA), which, however, is markedly limited due to its narrow time window and severe side effect such as hemorrhage [1, 2]. For neurodegeneration diseases such as Alzheimer's disease (AD), currently only symptomatic treatments are available, whereas disease-modifying treatments are still under development [3].

Recently, activation of endogenous neuroprotective mechanisms has emerged as a more promising strategy for the development of therapeutics against these neurological disorders. Neuroglobin (Ngb) is an oxygen-binding globin protein that was identified in 2000 [4]. Ngb has been demonstrated to be an endogenous neuroprotective molecule, as Ngb gene expression inversely correlated with the severity of histological and functional deficits in stroke and other related neurological disorders in both *in vitro* cell culture and *in vivo* animal models [5–9]. However, the underlying mechanisms of Ngb's neuroprotection remain largely not clarified [10, 11]. Mitochondria are key intracellular organelles that play prominent roles in energy metabolism, calcium homeostasis [12], redox signaling, reactive oxygen species (ROS) generation, and apoptosis-programmed cell death [13]. Neurons are particularly dependent on mitochondria because of their high energy demands [14], thus mitochondria dysfunction is correlated with a wide range of neurological disorders. Early studies have shown that Ngb expression is confined to metabolically active, oxygen-consuming cell types [15], therefore suggesting a functional relationship between Ngb and mitochondria. In this paper we briefly summarize the mechanisms of Ngb's neuroprotection that are related to mitochondria function and regulation.

## 2. Roles and Mechanisms of Ngb in Mitochondrial Function Related to Neurological Disorders

Accumulating evidence has demonstrated the neuroprotective roles of Ngb in a wide range of neurological disorders. For example, an *in vitro* study showed that antisense-mediated knockdown of Ngb rendered cortical neurons more vulnerable to hypoxia, whereas Ngb overexpression driven by CMV promoter in pcDNA vector, which yielded about 2-fold Ngb protein increase, protected cultured neurons against hypoxia [16]. Similar effect was observed in neuroblastoma cell line SH-SY5Y that Ngb overexpression by pEGFP-Ngb transfection (over 100-fold Ngb protein level increase, possibly due to low basal Ngb level) enhanced cell survival under anoxia or oxygen/glucose deprivation (OGD) [17]. In Ngb-transgenic animal studies, Ngb overexpression with over 2.7-fold Ngb level increase driven by CMV promoter [5] or a much higher Ngb level increase by chicken *β*-actin promoter [6] both ameliorated the severity of histological and functional deficits in mouse stroke models. Furthermore, antisense oligonucleotide-mediated endogenous Ngb knock-down deteriorated the outcome of focal cerebral ischemia in rats [8].

Ngb overexpression is also protective against beta-amyloid-induced neurotoxicity and Alzheimer phenotype *in vivo* in Ngb and APP (amyloid precursor protein) double-transgenic mice [7]. Furthermore, our study showed that Ngb overexpression (about 2.7-fold Ngb increase in Ngb-Tg mouse) protects retinal ganglion cells (RGC) against ocular hypertension and glaucomatous damage in mouse [9]. In these studies, mitochondrial functions including ATP production, ROS generation, and cell survival/death signaling have been significantly affected by the alteration of Ngb expression, which might be part of the mechanisms of Ngb's neuroprotection.

### 2.1. Ngb and Mitochondrial ATP Production

Although Ngb is an O_2_-binding protein, O_2_ transportation and supply to neurons may not be an important function of Ngb due to the high O_2_ binding rate and low O_2_ dissociation rate of Ngb, plus the relatively low level of Ngb protein in the brain (<1 *μ*M) [18, 19]. Nevertheless, the O_2_-binding property implies that Ngb may play a role in O_2_ sensing and cellular energy metabolism, that is, ATP production. The molecular machinery required for ATP production is mitochondrial electron transport chain (ETC), which is an assembly of electron donors and acceptors, namely, complex I, II, III, and IV, that reside in the cristae and matrix of mitochondria. ATP production starts from citric acid cycle using pyruvate as the substrate. The coenzymes nicotinamide adenine dinucleotide (NAD) and flavin adenine dinucleotide (FAD) [20] are reduced during the citric acid cycle, yielding NADH and FADH2, respectively, which supply electron and energy to ETC. The electron is passed along the cascade of complex I–IV and protons are pumped across the inner membrane to establish a proton gradient. The mitochondria membrane potential (MMP) generated by oxidative phosphorylation (OXPHOS) across the inner membrane is a vital feature of mitochondria and plays essential roles in mitochondrial functions such as ATP and ROS production [21]. ATP is finally synthesized by ATP synthase when the chemiosmotic gradient drives the phosphorylation of ADP [22].

Stroke is associated with great ATP loss. The drastic reduction in blood flow within the ischemic core area leads to a shortage in O_2_ and glucose supply and eventually reduced ATP production. ATP level of the ischemic core area falls markedly during the first 5 min or so of arterial occlusion and then stabilizes at levels approximately 15–30% of nonischemic tissue for at least the first 2 h of focal ischemia [23–25]. A similar pattern of energy alteration happens in the penumbral area, but the drop of ATP content is less severe. The ATP loss reflects the impaired mitochondrial oxidative metabolism [24] and is consistent with the increased lactate level in the penumbra area, since lactate is the fermentation product of pyruvate. Consistent with the reduced ATP level following ischemia, the capacity of mitochondria respiration is considerably decreased in both the core tissue and penumbra area [26, 27]. Reperfusion can transiently restore the mitochondrial respiratory function, which is then decreased at later times [26, 28]. The possible mechanisms that cause the failure of mitochondrial respiration include oxidative stress and cytochrome c (Cyt c) release from mitochondria to cytosol [29, 30].

Early experimental observations have suggested a close link between Ngb and mitochondria. Ngb is highly expressed in retina, and its distribution in photoreceptors correlates with the subcellular localization of mitochondria, that is, the plexiform layers and the inner segment which consume most of the retinal oxygen [31]. Later studies further showed the roles of Ngb in maintaining mitochondria function in response to insults. For example, Ngb overexpression by plasmid (pcDNA3-Ngb) transfection (2–8 fold Ngb protein increase) promoted cell survival against beta-amyloid toxicity and attenuated beta-amyloid-induced mitochondrial dysfunction in cultured PC-12 cells [32]. Ngb overexpression by chicken *β*-actin promoter in Ngb transgenic mouse also eliminated hypoxia-induced mitochondrial aggregation and neuron death [33]. Our lab has demonstrated that Ngb overexpression (about 2.7-fold Ngb protein increase in Ngb transgenic mouse) improved mitochondrial function in cultured mouse cortical neurons [34]. We showed that the rate of decline of ATP level was significantly ameliorated by Ngb overexpression at early time points after hypoxia/reoxygenation. In another study, elevation of Ngb expression by Ngb plasmid (pDEST40-Ngb) transfection (about 4-fold Ngb protein increase) in SH-SY5Y cell line led to a significant decrease in oxidative stress caused by H_2_O_2_ and an increase in the intracellular ATP level [35]. Furthermore, Ngb overexpression by plasmid (pDEST40-Ngb) transfection increased cell viability and inhibited hypoxia/reoxygenation-induced ATP loss in cultured human neuronal cells [36]. All of these data suggested a function of Ngb in preserving mitochondrial ATP production, either through preserving general mitochondrial function or specific influence on mitochondrial respiration; however, the exact mechanisms remain undefined.

Ngb protein was mainly localized in cytosol [37], which is supportive of its role in O_2_ binding and sensing and involvement in cell signaling, for example, its possible function as guanine-nucleotide-dissociation inhibitor (GDI) [38]. However, accumulating evidence has revealed that Ngb is not only localized in cytosol, but also is closely associated with mitochondria. For example, Hundahl et al. detected subcellular localization of Ngb in neuronal cytosol, mitochondria and nucleus using immunocytochemistry and electron microscopy [39]. Additional evidence includes the observed colocalization of Ngb with neuronal nitric oxide synthase (nNOS), an indirect suggestion of Ngb's association with mitochondria since nNOS is present in both cytosol and mitochondria [40]. We recently performed yeast two hybrid assay to identify Ngb-binding proteins, and one of them is cytochrome c1 (Cyc1), a subunit of mitochondria complex III [41]. In support of this finding, our recent study clearly demonstrated that Ngb is physically localized in the mitochondria of primary cultured cortical neurons using Western blot, immunocytochemistry, and immunoelectron microscopy, although the mitochondrial Ngb is only a small portion (~10% of total Ngb), and the major portion of Ngb is in cytosol (~90%) [42]. In this study, the specificity of anti-Ngb antibody was validated by preabsorption with recombinant Ngb and secondary antibody alone to ensure the reliability of our results. Additionally, a very recent study further confirmed that Ngb is localized inside the mitochondria of retinal neurons [43]. The authors treated the mitochondria fractions with proteinase K (PK) and showed that significant amount of Ngb was insensitive to PK-induced proteolysis, therefore indicating that Ngb was truly integrated into the mitochondria. They further showed that Ngb knockdown can reduce the activities of mitochondrial complexes I and III [43]. These studies suggest that Ngb might play a role in ATP production through binding with respiratory complexes and affecting their activities. 

Despite the previous evidence for Ngb's subcellular localization and association with mitochondria, Hundahl et al. [44] recently raised a critical point that a fully validated anti-Ngb antibody is essential in detecting Ngb's subcellular localization and function, and the most reliable validation method is to use Ngb-null mice, which is only available a short time ago [45]. In this regard, great cautions should be taken when trying to interpret the previously reported subcellular localization of Ngb detected by immunostaining. The physical and functional associations of Ngb with mitochondria may not be conclusive so far but should be further investigated using fully validated anti-Ngb antibody.

### 2.2. Ngb and Mitochondrial ROS Generation, Oxidative Stress

In addition to the putative role of Ngb in ATP production, it is possible that Ngb may also be involved in another aspect of mitochondria respiration, that is, ROS (reactive oxygen species) production. This hypothesis was supported by the fact that Ngb can bind to nitric oxide (NO) besides O_2_ [46]. ROS was produced at the end of mitochondrial respiration when a portion of electrons leak to oxygen through complex I and III, generating superoxide radical anion (superoxide anion,  O_2_^•  −^) [47]. The rate of superoxide anion production increases when the electron carriers harbor excess electrons, for example when oxidative phosphorylation is inhibited under pathological conditions. Superoxide anion can be converted to H_2_O_2_ by manganese superoxide dismutase (MnSOD) or CuZnSOD in the intermembrane space. A series of reactive species could be further derived from superoxide anion and H_2_O_2_, including reactive hydroxyl radical (OH^•^) and carbonate radical anion (CO_3_^•−^), which altogether make up a family of reactive oxygen species (ROS) [48]. Another reactive species, peroxynitrite (ONOO^−^), is produced by the reaction of superoxide anion with nitric oxide (NO) *in vivo *[49] or synthesized from hydrogen peroxide and nitrite [50] and can react with other molecules to make additional types of reactive nitrogen species (RNS). Rapid increase of ROS has been demonstrated in ischemic stroke, both during ischemia and reperfusion [51, 52]. ROS can initiate damage to nucleic acids, proteins, and lipids in both mitochondria and cytosol [53]. Proteins were damaged by ROS through oxidation or nitration of various amino acids side chains, generating glutamate and aminoadipic semialdehydes [54]. Moreover, several enzymes in the ETC have been shown to be inhibited by ROS, resulting in compromised ATP synthesis [55].

Under normal physiological conditions, ROS was maintained at a safe level by enzymatic or nonenzymatic antioxidant machineries. Major ROS-scavenging enzymes include the superoxide dismutase (SOD), catalase (CAT), glutathione peroxidase (GPX), and peroxiredoxins (Prx), while nonenzymatic antioxidants include ascorbic acid (vitamin C), tocopherols (vitamin E), and glutathione (GSH) [56]. Interestingly, ROS can act as signaling molecules to regulate the expression of antioxidant genes, providing a feedback regulation mechanism for ROS levels. For example, in cultured mouse muscle cell line, H_2_O_2_ exposure led to both gene transcription and enzymatic activity increase for antioxidant genes SOD, GPx, and CAT [57]. In addition, another study showed that antioxidant enzymes GPX and heme oxygenase-1 (HO-1) were upregulated by repetitive ischemia/reperfusion (I/R) in mouse heart, and this upregulation was dependent on ROS [58].

Ngb has been proposed to have a ROS scavenging function. Our previous study showed that Ngb overexpression (~2.7-fold increase in Ngb transgenic mouse) significantly reduced the generation of superoxide anion after hypoxia/reoxygenation in primary cultured mouse cortical neurons compared to wild-type-controls [34]. Additionally, other than O_2_ binding, Ngb can also bind NO [59] and can protect against NO-induced neurotoxicity [60], suggesting that Ngb may neutralize the neurotoxic effects of reactive nitrogen species (RNS), which may be another mechanism of Ngb's neuroprotection. Furthermore, Ngb overexpression (~2.6-fold increase in Ngb transgenic mouse) is associated with significantly reduced ROS/RNS production and lipid peroxidation in the CA1 region and reduced CA1 neuronal injury in a mouse model of ischemia-reperfusion injury [61]. However, the mechanisms of this effect remain unclear. It might be ascribed to direct binding between Ngb and these reactive species or the interaction between Ngb and mitochondrial respiration chain components, such as mitochondrial complex III, which was demonstrated by our recent studies [11, 42], but it could also be an indirect effect through a general improvement of mitochondrial function. A recent study using recombinant human Ngb (rhNgb) confirmed that Ngb has a direct antioxidant capacity and can efficiently scavenge a variety of free radicals, including the [2,2′-azino-di-(3-ethyl-benzthiazoline-6-sulfonic acid)] (ABTS) cation, superoxide anion, hydrogen peroxide, and hydroxyl radical [62]. The capacity of rhNgb in scavenging superoxide anion was less but comparable with equal amount of vitamin C (Vc) (from 2.5–12.5 *μ*g/mL) but far superior than GSH. Furthermore, rhNgb's capacity in scavenging hydrogen peroxide was even higher than Vc at 10 *μ*g/mL [62].

Antioxidant treatments have been investigated as potential therapeutics for stroke. The compounds with ROS scavenging capabilities such as lipoic acid and the glutathione precursor, NAC (n-acetyl-cysteine), were reported to reduce infarct volume in animal stroke models [63–65]. Other potent free radical scavengers include NXY-059, edaravone, and resveratrol, which have been proved to protect against stroke and brain trauma in animal models and are being tested in clinical trials [66–69]. Similarly, ATP restoration could also be targeted, maybe indirectly, for developing therapies against stroke. For example, nicotinamide was protective in a mouse model of ischemia reperfusion by providing a reserve of NAD+ and restoring ATP level [70]. Another study showed that histone deacetylase (HDAC) inhibitors protect mouse optic nerve from OGD-induced damage, partially through preserving ATP levels [71]. Additionally, coenzyme Q10 (CoQ10), the electron acceptor of complex I and II in ETC, is neuroprotective in a rat model of cerebral ischemia, probably through conserving ATP production and antioxidant property [72]. Ideally, it would be more efficient for a treatment to target more than one protection mechanisms. In this regard, since Ngb has multiple protective mechanisms including preserving ATP and scavenging ROS, the development of endogenous Ngb upregulation strategy might be a potentially more effective therapy against neurological disorders, which warrants further investigation [11].

### 2.3. Mitochondria-Mediated Cell Death Signaling and Ngb

Other than the roles of Ngb in preserving mitochondrial ATP production and scavenging ROS, Ngb has also been hypothesized to be a signaling molecule. For example, it was found that ferric human Ngb (met-Ngb) binds to the GDP-bound state of G protein  *α*  subunit (G*α*) and exerts guanine-nucleotide dissociation inhibitor (GDI) activity [38]. Ferric Ngb inhibits the exchange of GDP for GTP, thus prevents the G*α*  subunit from binding to the G*βγ* complex and activates the downstream signal transduction pathway, which is protective against oxidative stress [38, 73].

Mitochondria play key roles in cell death and survival signaling in response to injuries. The direct effectors of mitochondria membrane disruption include a group of prodeath Bcl-2 family proteins such as Bax, Bak, Bid, Bim, Bad, and PUMA, among others. Bax and Bak directly cause mitochondrial membrane disruption via channel formation in mitochondrial outer membrane. Bid and PUMA function in facilitating Bax and Bak channel formation, whereas Bad and Bim inhibit prosurvival Bcl-2 and Bcl-xL [74]. 

After mitochondria membrane disruption, proapoptotic molecules such as Cyt c and apoptosis-inducing factor (AIF) are released into cytosol and initiate caspase-dependent and -independent cell death, respectively. Released Cyt c in cytosol initiates the assembly of apoptosome by binding with Apaf 1, which in turn activates caspase 9. Caspase 9 goes on to activate caspase 3 and caspase 7 [75]. In caspase-independent apoptosis, AIF translocates into nucleus, where it initiates the chromosomes condensation and DNA fragmentation, the key step of apoptosis [76]. Other proapoptotic proteins released from mitochondria include procaspases, EndoG, Smac/DIABLO, and Omi/HtrA2 [77].

Mitochondrial ROS is also actively involved in cell death signaling pathways. The early evidence for the involvement of mitochondrial ROS in cell death arose from the study of TNF-*α*-induced cytotoxicity [78]. Mounting evidence later from studies using antioxidants or ROS-overproduction approaches has demonstrated the central roles of ROS in cell death signaling pathways, including programmed cell death (PCD) [79].

Ngb may play a regulatory role in neuronal signaling pathways in response to insults such as hypoxia. Khan et al. [33] have shown that Ngb overexpression in primary neuron culture from Ngb-transgenic mouse diminished hypoxia-induced microdomain polarization and mitochondrial aggregation, the early responses of neurons to death stimuli. Subsequently, Cyt c is released from mitochondria to cytosol, which is generally believed to be caused by mitochondrial permeability transition pore (mPTP) opening [80], followed by activation of caspase-dependent or -independent apoptosis pathways. Studies in our lab have shown that Ngb overexpression by AAV-Ngb transduction (about 4-fold Ngb level increase) is correlated with reduced mPTP opening and decreased Cyt c release in primary cultured mouse cortical neurons after oxygen/glucose deprivation (OGD) and reoxygenation (unpublished data). This suggests an inhibitory effect of Ngb in OGD-induced mPTP opening, which could be one of the mechanisms of Ngb neuroprotection. The reduced Cyt c release by Ngb overexpression may be partially attributed to the inhibition of mPTP opening by Ngb. However, other mechanisms may also be involved. For example, an *in vitro* biochemistry study showed that ferrous Ngb can rapidly reduce ferric Cyt c, converting ferric Cyt c to ferrous Cyt c [81]. Since Cyt c released from mitochondria is predominantly in the ferric form [82], and only ferric Cyt c, but not ferrous Cyt c, was reported to be active in initiating apoptosis [83], thus ferrous Ngb may prevent apoptosis initiation by reducing ferric Cyt c. Furthermore, computational modeling confirmed that the binding of Ngb to Cyt c and the subsequent redox reaction can block caspase 9 activation [84, 85]. It is possible that Ngb-Cyt c binding is also causative for decreased Cyt c release from mitochondria, which remains to be further investigated.

It should be emphasized that the redox state of Ngb not only is critical in regulating Cyt c-mediated apoptosis but also may have significant implications in other functions of Ngb. For example, ferrous Ngb is more favorable in NO scavenging than ferric Ngb [46]. It is therefore important to maintain the redox cycling of Ngb. Although an NAD(P)H-dependent Ngb-reductase activity has been detected in human brain tissue homogenates [86], the enzyme(s) responsible for this activity has not been identified [87]. More advanced study about the Ngb reductase system is highly warranted in the future, which would greatly enhance our understanding of the regulation mechanisms of Ngb function.

Calcium is a key signaling molecule for many cellular functions including apoptosis [88]. A major source of cytosolic calcium is endoplasmic reticulum (ER) [89]. Calcium is involved in regulating mitochondrial morphology and release of proapoptotic proteins. Upon death stimuli, calcium can be released from ER and fluxed into mitochondria, resulting in mitochondria swelling and fragmentation and subsequent Cyt c release [90]. Interestingly, it was reported that Cyt c released from mitochondria at the initiation of apoptosis can translocate into ER and bind to inositol (1,4,5) trisphosphate receptor (InsP_3_R); this binding leads to more calcium release from ER and increased cytosolic calcium level, which in turn results in coordinate release of Cyt c from all mitochondria and amplifies the apoptosis signal [91]. Ngb may also play a role in apoptosis by regulating cytosolic calcium level in response to death stimuli. It has been reported that Ngb overexpression by plasmid transfection (pDEST40-Ngb) significantly blocked hypoxia/reoxygenation-induced cytosolic calcium level increase in cultured neuronal cell lines [36]. This effect could be either through regulating membrane transporters or calcium release from ER, which is worth being further investigated.

**Figure 1:**
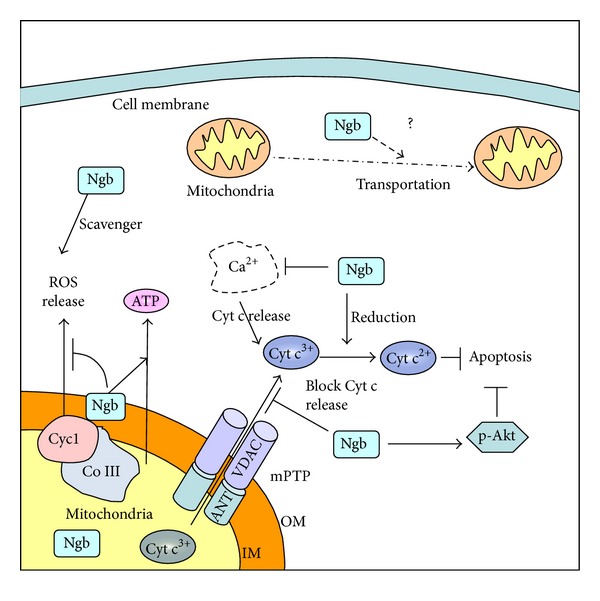
Potential mitochondrial mechanisms of Ngb neuroprotection. Ngb may be neuroprotective by preserving mitochondrial ATP production and scavenging ROS. Ngb may bind to VDAC, inhibit mPTP opening after OGD, and then block Cyt c release from mitochondria and the subsequent apoptosis. Ferrous Ngb can convert ferric Cyt c to ferrous Cyt c and thus prevent ferric Cyt c-induced apoptosis initiation. Ngb may ameliorate injury-induced calcium influx, therefore inhibiting calcium-induced amplification of Cyt c release and apoptosis. Ngb may also inhibit apoptosis by activating p-Akt. Finally, Ngb might have some effect in mitochondria transportation. OM: mitochondria outer membrane and IM: mitochondria inner membrane.

Furthermore, Ngb may prevent apoptosis by indirectly modulating apoptosis regulators. For example, Ngb overexpression by plasmid (pDEST40-Ngb) transfection in SH-SY5Y cells protects against H_2_O_2_ injury by sustained activation of mito-K_ATP_ channel and Akt phosphorylation [35]. Phosphorylated Akt (p-Akt) inhibits the release of AIF and Cyt c, thereby inhibiting apoptosis [92], and thus the effect of Ngb in p-Akt may be another way of Ngb in regulating apoptosis. The possible involvements of Ngb in ATP production, ROS scavenging, and mitochondria-mediated apoptosis signaling were summarized in Figure 1.

A lot of studies have shown that strategies targeting apoptosis are neuroprotective in various animal stroke models [77]. For example, specific inhibitors for caspase-3 and caspase-9 ameliorated brain tissue loss and improved neurological outcomes in rat or mice stroke models [93–95]. It is reasonable to assume that a strategy targeting upstream regulators of mitochondria-mediated cell death pathway would provide better neuroprotection than targeting downstream regulators. One important upstream regulator is c-Jun N-terminal kinase (JNK). JNK can phosphorylate the scaffolding protein 14-3-3 and lead to the translocation of Bax into mitochondria [96], which further results in inhibition of prosurvival Bcl-xL and Bcl-2 [97]. JNK inhibition using pharmacological inhibitor [96] or the small peptide inhibitor D-JNKI-1 [98] has shown prolonged neuroprotection for up to 14 days of reperfusion in animal models of focal ischemia. Based on the potential effect of Ngb in inhibiting apoptosis, targeting apoptosis inhibition by Ngb upregulation may be a more effective strategy for treatment of stroke and related neurological disorders, since Ngb may confer neuroprotection via multiple mechanisms including preserving ATP and scavenging ROS as well. A possible strategy for this purpose is to screen for endogenous Ngb upregulating compounds as potential therapies against brain injuries including stroke [11].

## 3. Molecular Interactions between Ngb and Mitochondria

Other than the proteins involved in normal mitochondrial functions, such as the protein components of electron transfer chain (ETC), mitochondria also harbor numerous proteins that are originally localized in cytoplasm but are translocated to mitochondria in response to death stimuli. Some of these are apoptosis signaling proteins like the prodeath Bcl-2 family members [74]. Additionally, emerging data show that mitochondria also host endogenous neuroprotective molecules such as Ngb, which might also contribute to the neuroprotection of Ngb [42].

We previously described that Ngb plays important roles in mitochondrial functions such as ATP production, ROS generation, and apoptosis signaling. To further dissect the molecular mechanisms of Ngb neuroprotection, our laboratory recently performed a screening for the protein interaction partners of mouse Ngb using yeast two-hybrid assay. We identified several Ngb-binding proteins, including Na/K ATPase beta 1, cytochrome c1 (Cyc1), ubiquitin C, voltage-dependant anion channel (VDAC), and a few more [41]. Interestingly, among these Ngb-binding proteins, VDAC and Cyc1 are mitochondrial proteins that are biologically important for neuronal function and survival. Cyc1 is a subunit of the mitochondria complex III, which is critical for mitochondrial ATP production and the generation of superoxide anion [99]. Cyc1 also plays pathological roles in response to oxidative stress [100] and regulates hypoxia-inducible-factor-1 (Hif1) activation induced by hypoxia. VDAC is a critical regulator of mitochondria permeability transition pore (mPTP) opening [101]. As a support for Ngb binding with mitochondrial proteins, our recent study clearly demonstrated that Ngb can be localized in mitochondria, and this localization was increased by OGD/reoxygenation [42]. Furthermore, Ngb overexpression is correlated with increased mitochondrial distribution of Ngb, suggesting the mitochondrial localization of Ngb may be important for neuroprotection. The mitochondrial localization of Ngb was further confirmed by another recent study in retinal neurons [43]. However, the detailed function of Ngb in mitochondria and its binding with mitochondrial proteins requires further investigation. The binding of Ngb with Cyc1 and complex III might be one of the mechanisms of Ngb's role in mitochondrial respiration and ATP production. Ngb binding with VDAC might have some effect in regulating mPTP, as we have found that Ngb overexpression can inhibit OGD-induced mPTP opening and subsequent Cyt c release (unpublished data). These potential mechanisms of Ngb neuroprotection were summarized in Figure 1.

## 4. Putative Involvement of Ngb in Mitochondrial Dynamics?

Mitochondria are remarkably dynamic organelles as they undergo repeated fission and fusion to interchange their contents. Mitochondria are also actively transported to subcellular sites where a high level of energy is required. Moreover, the quality of mitochondria is maintained through mitophagy in which defective mitochondria are selectively degraded. Dysfunction in mitochondrial dynamics is widely implicated in neurodegenerative diseases such as Parkinson's [102]. Since Ngb plays a role in mitochondrial energy production and can affect mitochondrial aggregation induced by hypoxia [33], it may be worth investigating the possible roles of Ngb in mitochondrial dynamics.

### 4.1. Brief Introduction of Mitochondrial Dynamics

Proper fission and fusion are required for maintaining normal mitochondrial function. Mitochondria fission is mediated by two key proteins, dynamin-related protein 1 (Drp1) and Fis1 [103], while mitochondrial fusion requires two families of dynamin-like proteins, Mfn1/Mfn2 and OPA1 [104]. Emerging evidence has linked mitochondria fission/fusion defects with neurodegenerative diseases [105, 106]. Inhibition of mitochondrial fission by knockdown of Drp1 or overexpression of Mfn1 mitigated NO-induced neuronal cell death, suggesting a role of mitochondrial fission in neuron death [106]. A recent study revealed that Drp1 and Opa1 were both upregulated in the ischemic penumbra but decreased in the ischemic core area after transient middle cerebral artery occlusion (tMCAO) in mice [107], suggesting a continuous mitochondrial fission and fusion in the salvageable ischemic penumbra.

It is crucial for mitochondria to transport to subcellular regions such as presynaptic terminals where high energy is demanded [108]. Mitochondria are transported along microtubule tracks, which are driven by ATP-dependent “motor” proteins, mainly the kinesin family members and dynein [109, 110]. Kinesins do not directly bind with mitochondria but through the adaptor proteins including TRAK1, TRAK2 and MIRO1, MIRO2 [111, 112]. Mitochondrial transport and spatial distribution in neurons are directly correlated with synaptic activity and neuron viability [113]. For example, hypoxia/reoxygenation in cultured cortical neurons impaired mitochondrial movement and morphology [114].

Proper and timely degradation of damaged and aged mitochondria is crucial for mitochondrial quality control. Dysfunctional mitochondria are cleared by mitophagy, a process that selectively eliminates mitochondria by autophagy [115]. The key mediators of mitophagy include Parkin and PINK1 (PTEN-induced putative kinase 1) [116–118]. It was recently reported that ischemic preconditioning induced Parkin translocation to mitochondria and increased ubiquitination in cardiomyocytes, therefore promoting mitophagy [119]. Parkin knockout abolished the ischemic preconditioning-induced mitophagy and the cardioprotection effect as well [119]. Mitophagy is also involved in neurodegeneration. One example is in degenerating Purkinje neurons, a common feature of inherited ataxias in humans and mice, mitophagy was found to be abnormally enhanced, suggesting an important role of the properly regulated mitophagy in neuronal function [120].

### 4.2. Potential Involvement of Ngb in Mitochondria Dynamics? 

One early study showed that Ngb overexpression can eliminate hypoxia-induced mitochondrial aggregation [33]. Mitochondrial aggregation is an event upstream of Cyt c release from mitochondria during apoptosis, and mitochondria transportation is very likely to be involved in this process [121], and thus the study by Khan et al. [33] suggested that Ngb may also play a role in mitochondria transportation under hypoxic/ischemic conditions. As an indirect supporting evidence of Ngb's role in mitochondria transport, Antao et al. found that Ngb overexpression can ameliorate H_2_O_2_-induced actin condensation, suggesting a potential function of Ngb in maintaining cell membrane integrity [35]. Since actin cytoskeleton is required for short-term mitochondrial movement and mitochondrial immobilization in neurons [122], this data further suggested an indirect role of Ngb in mitochondrial transportation. Further studies will be valuable to investigate the function of Ngb in mitochondrial dynamics.

## 5. Summary 

In summary, Ngb is an endogenous neuroprotective molecule against stroke and related neurological disorders, but the neuroprotection mechanisms remain largely undefined. Mitochondria are key players in neuronal death and survival determination in stroke and related neurological disorders, affecting multiple pathophysiological processes including energy metabolism, cellular homeostasis, and cell death signaling pathways. It has been demonstrated that Ngb preserves mitochondria ATP production, reduces ROS generation, and participates in mitochondria-mediated cell death signaling. However, the detailed molecular interactions between Ngb and mitochondrial proteins remain to be further elucidated, which will be beneficial in understanding the mechanisms of Ngb's neuroprotection and development of Ngb and mitochondria-targeted therapeutics against stroke and related neurological disorders.
